# Increased Finger-Tapping Related Cerebellar Activation in Cervical Dystonia, Enhanced by Transcranial Stimulation: An Indicator of Compensation?

**DOI:** 10.3389/fneur.2019.00231

**Published:** 2019-03-15

**Authors:** Thorsten M. Odorfer, György A. Homola, Martin M. Reich, Jens Volkmann, Daniel Zeller

**Affiliations:** ^1^Department of Neurology, University of Würzburg, Würzburg, Germany; ^2^Department of Neuroradiology, University of Würzburg, Würzburg, Germany

**Keywords:** cervical dystonia, functional MRI, cortical excitability, transcranial magnetic simulation (TMS), continuous theta burst stimulation (cTBS), motor-evoked potentials (MEP), cortical silent period

## Abstract

**Background:** Cervical dystonia is a movement disorder causing abnormal postures and movements of the head. While the exact pathophysiology of cervical dystonia has not yet been fully elucidated, a growing body of evidence points to the cerebellum as an important node.

**Methods:** Here, we examined the impact of cerebellar interference by transcranial magnetic stimulation on finger-tapping related brain activation and neurophysiological measures of cortical excitability and inhibition in cervical dystonia and controls. Bilateral continuous theta-burst stimulation was used to modulate cerebellar cortical excitability in 16 patients and matched healthy controls. In a functional magnetic resonance imaging arm, data were acquired during simple finger tapping before and after cerebellar stimulation. In a neurophysiological arm, assessment comprised motor-evoked potentials amplitude and cortical silent period duration. Theta-burst stimulation over the dorsal premotor cortex and sham stimulation (neurophysiological arm only) served as control conditions.

**Results:** At baseline, finger tapping was associated with increased activation in the ipsilateral cerebellum in patients compared to controls. Following cerebellar theta-burst stimulation, this pattern was even more pronounced, along with an additional movement-related activation in the contralateral somatosensory region and angular gyrus. Baseline motor-evoked potential amplitudes were higher and cortical silent period duration shorter in patients compared to controls. After cerebellar theta-burst stimulation, cortical silent period duration increased significantly in dystonia patients.

**Conclusion:** We conclude that in cervical dystonia, finger movements—though clinically non-dystonic—are associated with increased activation of the lateral cerebellum, possibly pointing to general motor disorganization, which remains subclinical in most body regions. Enhancement of this activation together with an increase of silent period duration by cerebellar continuous theta-burst stimulation may indicate predominant disinhibitory effects on Purkinje cells, eventually resulting in an inhibition of cerebello-thalamocortical circuits.

## Background

Cervical dystonia (CD) is a movement disorder leading to involuntary muscle contractions which cause repetitive and twisting head movements and abnormal, sometimes painful head postures (1). Dystonic disorders have been regarded as psychogenic diseases for decades (2) before pathophysiological research provided evidence for underlying basal ganglia dysfunction (3, 4). Only over the last years, a growing body of evidence points to the cerebellum (CRB) as an important node in dystonia pathophysiology (5–8). Most of this evidence originates from magnetic resonance imaging (MRI) studies, particularly from advanced techniques like functional (9, 10) and resting-state MRI (11), voxel-based morphometry (VBM) (12–15), or probabilistic tractography (16) in different cohorts of dystonia patients. While this leaves little doubt as to cerebellar involvement in dystonic disorders, the exact nature of this involvement remains unclear. In the case of cervical dystonia, functional imaging faces additional challenges: While brain activation associated with dystonic head movement would be of particular interest, data acquisition requires subjects to keep the head still. As the interpretation of task-free functional imaging studies in CD may be ambiguous (17, 18), simple hand motor tasks have been used instead to study activation patterns in functional MRI (fMRI). Although clinically non-dystonic, such hand movements have been shown to be associated with altered activity in ipsilateral putamen, insula and cingulate cortex (19) as well as caudate nucleus, putamen and thalamus (20). In an upper limb force task, increased severity of CD was associated with decreased functional activity of the somatosensory cortex and increased activity of CRB (21). Only recently, Prudente et al. used a new paradigm to assess the functional imaging correlate of isometric head movements (22). They found increased activation of the anterior CRB during constant tension on muscles rotating the head into the pathological direction of torticollis (22). However, while fMRI is able to reveal brain activity associated with a certain condition, the technique is unable to discriminate pathophysiological from compensatory activation. Here, additional neurophysiological approaches like repetitive transcranial magnetic simulation (rTMS) can prove useful (23): By interfering with neuronal processes of a specific brain area, the functional role of this region can be probed (24). In this way, for instance, rTMS over the premotor cortex has been shown to improve symptom severity in CD patients (25). Moreover, a significant reduction of the Toronto Western Spasmodic Torticollis Rating Scale (TWSTRS) score following a 2 week cerebellar continuous theta-burst stimulation (cTBS) treatment has been reported (26). However, similar clinical improvement of CD has recently been described in a study applying cerebellar intermittent TBS (iTBS) over 10 working days (27), along with an improved performance in the pegboard task, i.e., an enhancement of motor function in a non-dystonic body part (27). From a mechanistic point of view, the results of these two studies appear conflicting: Given their opposite impact on excitability at the primary motor cortex (28), one might not expect that both stimulation protocols can induce clinical improvement when applied to the cerebellum. However, as cerebellar physiology and cytoarchitecture is largely different from the motor cortex, effects of cTBS on M1 may not easily be transferred one-to-one to the CRB. Therefore, rather than a dichotomic issue, the behavioral impact of cerebellar TBS might be considered a net effect of various neuromodulative effects of different direction.

In the present study, we applied a complementary approach to challenge the role of CRB in CD: First, we examined functional MRI (fMRI) brain activation during a simple finger tapping task along with neurophysiological measures of cortical excitability in CD patients as compared to healthy controls. Second, we assessed the effects of an excitability-modulating TMS protocol at the lateral CRB on (i) finger-tapping associated brain activation in fMRI, (ii) measures of cortical excitability, and (iii) clinical scores of CD severity. Changes in physiological and/or clinical measures were anticipated to allow an informed interpretation of fMRI data later-on.

## Methods

### Participants

Sixteen patients (7 females) with idiopathic cervical dystonia (CD) were recruited from our outpatient clinic for movement disorders. Neurological or psychiatric conditions other than CD led to exclusion from the study. All CD patients were treated with botulinum neurotoxin injections on a regular basis. The experiments were scheduled at an interval of at least 10 weeks from the last injection, with no or minor treatment effects remaining as judged both by the experimenter and by the patient. In addition, a control group (CTRL) of 16 healthy volunteers matched for age and sex (6 females) was recruited. Handedness was determined by a modified version of the Edinburgh Handedness Inventory (29). The protocol conformed to the principles of the declaration of Helsinki and was approved by the Ethics Committee of the Medical Faculty at the University of Würzburg. All participants gave their written informed consent for participation in the study.

### Study Design

**Figure 1:**
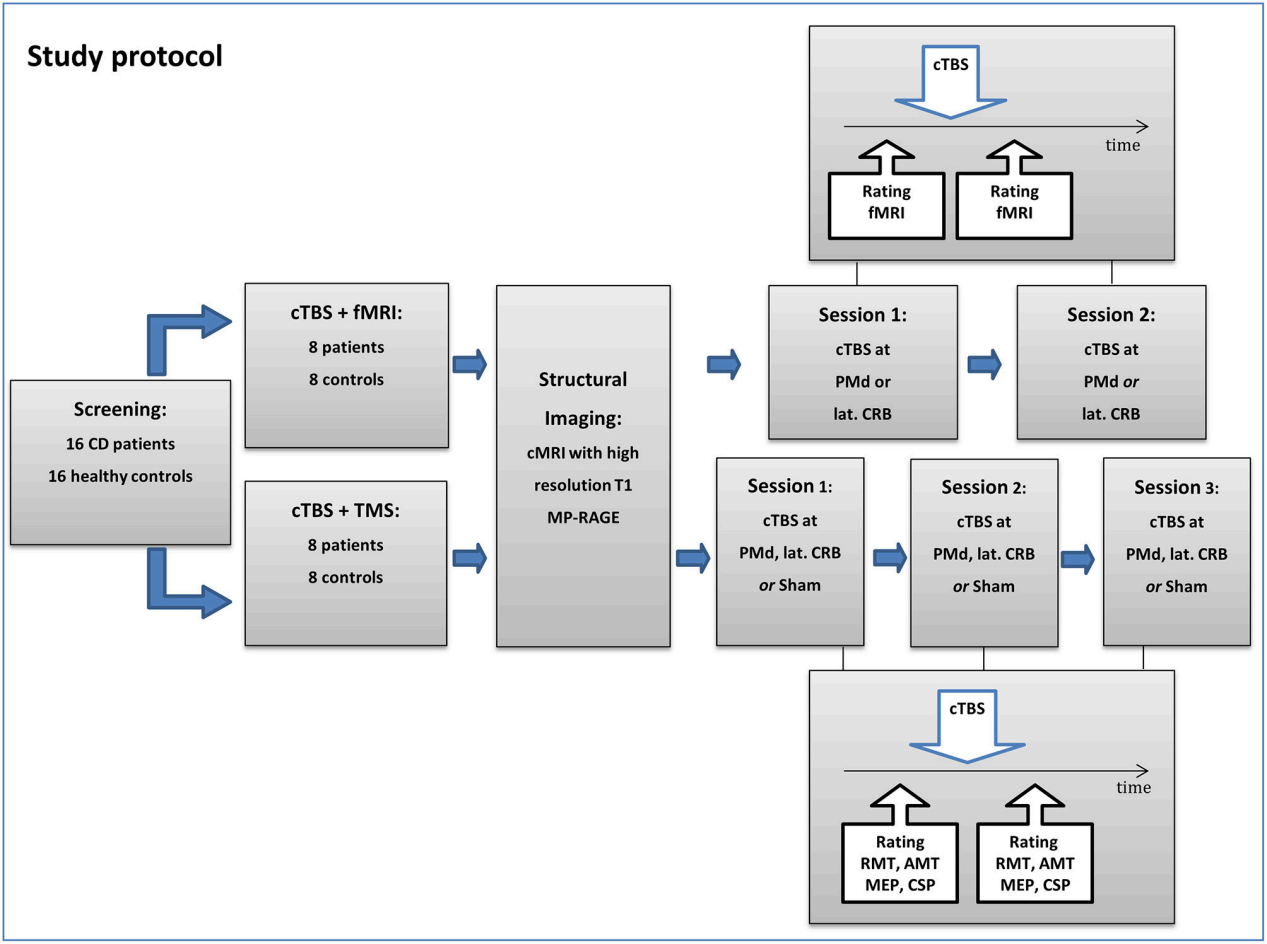
Flowchart of the study protocol. CD, cervical dystonia; cTBS, continuous theta-burst stimulation; TMS, transcranial magnetic stimulation; fMRI, functional magnetic resonance imaging; PMd, dorsal premotor cortex; lat. CRB, lateral cerebellum; RMT, resting motor threshold; AMT, active motor threshold; MEP, motor-evoked potential; CSP, cortical silent period.

Participants were randomized to two arms of the study (fMRI or TMS), with eight CD patients and eight controls per arm (Figure 1). In the TMS arm, participants underwent an electrophysiological work-up before and after cTBS at dorsal premotor cortex (PMd) and CRB, respectively, or sham stimulation (three sessions). In the fMRI arm, brain activation during simple finger tapping was assessed before and after cTBS at PMd or CRB (two sessions). In support of feasibility, the fMRI arm did not comprise an additional sham condition to reduce the single patient's burden within the study. The reason for using two experimental groups, rather than doing all experiments in one group, was the long total duration of five sessions which may overtax the compliance of participants [see also (30)].

### TMS and EMG Recording

All participants received high resolution MRI including T1-weighted (T1w) 3D MP-RAGE sequences (1 mm isotropic) to allow the localization of cortical regions by neuro-navigation (Brainsight, Rogue Research, Montreal, Canada). TMS was applied by a MC-B70 double coil connected to a MagPro X100 stimulator (Medtronic A/S 2140 Skovlunde, Denmark).

Electromyography (EMG) was recorded from first dorsal interosseous muscle (FDI) via surface cup electrodes with the reference placed over the metacarpophalangeal joint of the index finger. Signals were amplified using a differential amplifier (CED 1902, Cambridge Electronic Design, Cambridge, UK) and bandpass-filtered between 1 and 200 Hz. EMG signals were sampled at 5,000 Hz and digitized by an analog-converter (CED 1401 plus, Cambridge Electronic Design, Cambridge, UK).

The left motor hotspot (M1), defined as the optimal position for eliciting motor-evoked potentials (MEPs) in the right FDI muscle, was localized both functionally (TMS) and according to the landmarks described previously (31), with excellent congruence of the two. PMd was considered to be represented in the posterior part of the middle frontal gyrus, which was located around 2 cm anterior and 1 cm medial to the motor hot spot (32, 33). CRB was marked 3 cm lateral and 1 cm inferior to the inion (31, 34–36). Targeting M1 and PMd, the coil was held in a 45° angle to the sagittal plane with the handle in backward direction, while during cerebellar stimulation, the handle pointed upwards.

Resting motor threshold (RMT) was defined as the lowest stimulation intensity evoking MEP amplitudes of at least 50 μV in 5 out of 10 trials (monophasic pulse-shape). Active motor threshold (AMT) was determined during voluntary FDI activation at about 20% of maximal innervation (visual feedback) and defined as the lowest stimulation intensity evoking MEP amplitudes of at least 200 μV in 5 out of 10 attempts (biphasic pulse-shape).

### Continuous Theta-Burst Stimulation (cTBS)

cTBS was applied at 80% AMT (biphasic pulse shape) for a total duration of 40 s (total amount of 600 pulses) (28). Cerebellar stimulation was applied bilaterally (left side first, 60 s break between stimulations), while unilateral stimulation of the left PMd and unilateral cerebellar SHAM stimulation (20% AMT, outer edge of the TMS coil touching the back of the head) served as control conditions.

### fMRI Arm

fMRI (Magnetom Trio, Siemens, Munich, Germany) data [EPI, 3 mm isotropic, repetition time (TR) = 3,000 ms, echo time (TE) = 30 ms, 164 volumes] were acquired during a straightforward tapping task of the right index finger and thumb before and after cTBS. Via a simple block design paradigm (plus and minus signs) visually presented with OLED goggles [NordicNeuroLab AS (NNL), Bergen, Norway] patients were instructed to press buttons on a response grip (NNL) in a moderate frequency or to rest for the same duration of 30 s. The two conditions were run equally in a randomized order over a total time frame of 8 min. Feedback data was recorded with high accuracy (Presentation, Neurobehavioral Systems Inc., Berkeley, CA, USA.) To minimize artifacts due to head movements, the participant's head was properly fixed during image acquisition.

### TMS Arm

The MEP amplitude (mean of 30) at 130% RMT was taken as an estimate of corticospinal excitability. The CSP duration (mean of 10) as recorded during voluntary FDI pre-innervation (about 20% of maximal innervation) at 150% AMT (biphasic stimulation) was taken as a measure of cortical inhibitory mechanisms. Neurophysiological measures were recorded in the same sequence (RMT–MEP–AMT–CSP) before and after cTBS intervention.

### Clinical Assessment

Clinical severity of CD was rated on the motor subscale of the TWSTRS (37) and the TSUI scale (38) in a blinded manner by providing standardized video sequences of CD examination to an experienced clinical investigator uninvolved in the experiment. In addition, CD patients were asked to rate their personal impression of symptom improvement or deterioration after cTBS by using the Clinical Global Impression Improvement subscale CGI-I (39).

### Data Analysis

First level and group analysis of the fMRI data was carried out with FEAT, part of the FMRIB Software Library (FSL v5.0, FMRIB, Oxford, UK) (40, 41) (FMRIB Software Library). Fieldmap-based distortion-correction was applied to unwarp the data to increase registration accuracy. MCFLIRT was applied for motion estimation and correction (40). Finger tapping feedback data was added as an additional event variable to account for motor activation and variance. A 2 × 2 × 2 design was set up to test for site and group differences and also to verify that there has been no significant baseline variance between runs on different days. A whole brain correlation analysis was performed with cluster thresholding to correct for multiple comparisons. This method is based on Gaussian random field theory and is more sensitive to activation than a voxel based thresholding and is also less overly-conservative with respect to the familywise error rate than the Bonferroni correction (42).

GraphPad Prism (GraphPad Software, San Diego, CA, USA) was used for statistical analyses of TMS data. MEP amplitudes were measured peak-to-peak and averaged. CSP duration was determined by the time interval from MEP onset to the restarting point of EMG activity with 50% amplitude of pre-MEP level. We tested for normality using the Anderson-Darling-Test. In case of normal distribution, baseline TMS data were compared by two-tailed *t*-tests, otherwise by non-parametric Mann-Whitney-U test. Repeated measures two-way ANOVA was applied to compare between the three stimulation conditions within each group, and Sidak's multiple comparisons test was used for *post-hoc* analysis. Effects were considered significant if *p* < 0.05. If not stated otherwise, all values are given as mean ± standard deviation (SD).

## Results

Demographics and clinical baseline data of CD patients are shown in Table 1.

**Table 1:** Clinical characteristics of patients.

**Patient no**.	Age^*^	Age of onset^*^	**Dominant pattern**	**Second pattern**	**TWSTR baseline**	**TSUI-score baseline**
1	41–45	31–35	LC right	TC left	14	3
2	45–50	41–45	TC left	LS right	15	5
3	51–55	11–15	DHT	LC left	15	6
4	55–60	31–35	TC left	SE left	19	5
5	61–65	16–20	DHT	TC right	22	13
6	41–45	35–40	DHT	TC left	16	4
7	41–45	41–45	TC left	LC left	21	6
8	45–50	21–25	TC left	DHT	21	9
9	61–65	56–60	TC left	LC right	19	3
10	36–40	26–30	RC	DHT	17	4
11	51–55	35–40	DHT	TC left	16	8
12	61–65	55–60	TC left	LC right	20	6
13	51–55	51–55	TC right	DHT	24	13
14	46–50	41–45	TC left	DHT	21	6
15	51–55	51–55	LC right	SE right	22	10
16	46–50	41–45	DHT	LC left	18	5
Means	51.9^**^	38.5			18.8	6.6
± SD	7.5	13.5			3.0	3.2

* *presented in age of range in order to avoid providing indirectly identifiable patient data*.

** *for comparison, healthy control group: mean age 45.0 ± 15.6 years (p = 0.125)*.

*TWSTRS, Toronto Western Spasmodic Torticollis Rating Scale; LC, laterocollis; TC, torticollis; LS, lateral shift; DHT, dystonic head tremor; SE, shoulder elevation; RC, retrocollis; AC, anterocollis*.

### fMRI

**Figure 2:**
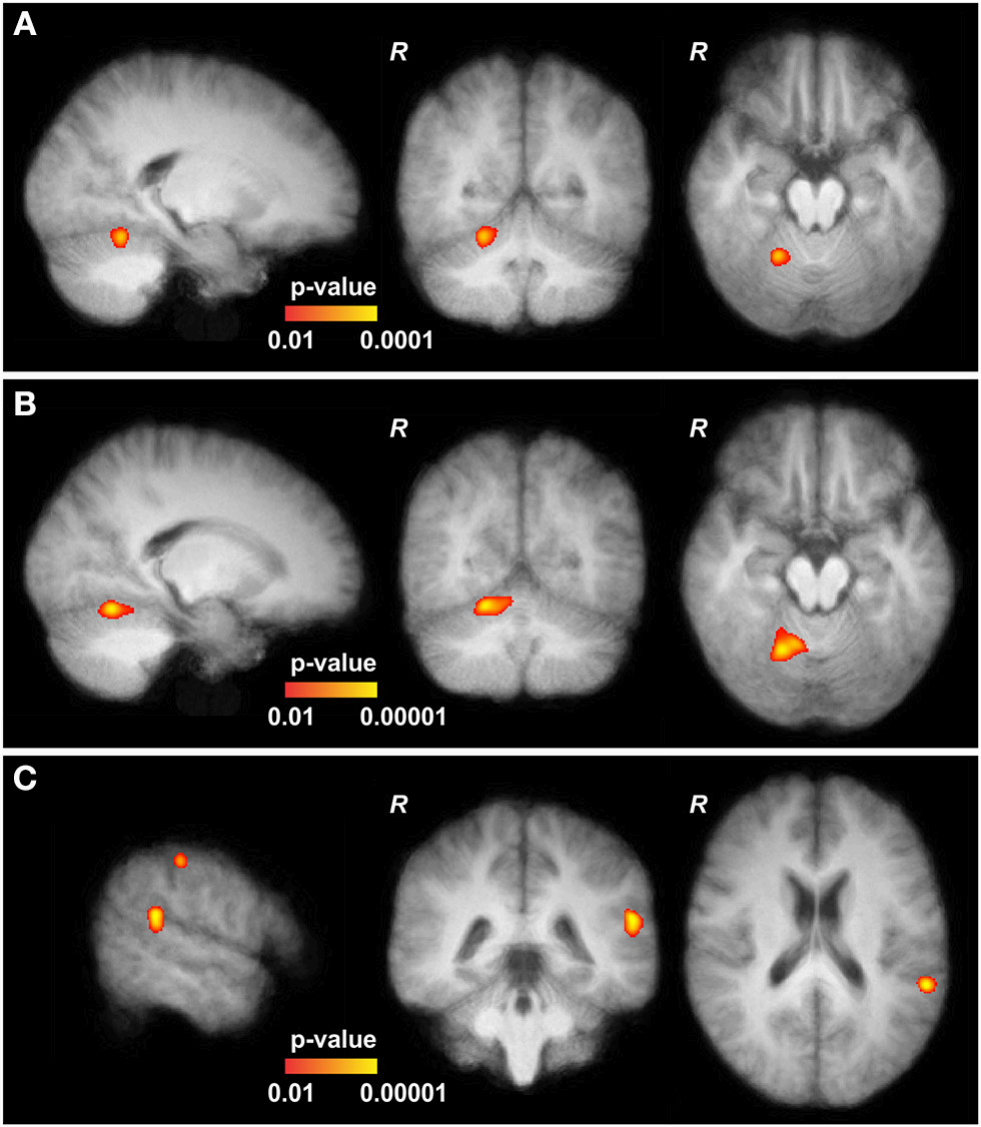
Functional MRI data of cervical dystonia (CD) patients**. (A)** At baseline, the upper part of the cerebellum (CRB) of CD patients showed slightly increased activation in comparison to controls (MNI152 21, −54, −18). **(B)** After continuous theta-burst stimulation (cTBS), main and significantly increased activations in CD patients are shown in the upper part of the right CRB (MNI152 19, −59, −16, adjacent to the baseline results, further pronounced). **(C)** Two other significantly elevated activations were found adjacent to the gyrus angularis (MNI152 −57, −42, 21) and the postcentral sulcus (MNI152 −55, −27, 48). All depicted activations are overlayed on the average coregistered and linearly transformed brains of the subjects. Some moderate but significantly elevated activations in the left primary motor and primary somatosensory cortex and the left premotor cortex are not shown. Comparison with patients at baseline and after stimulation of the left dorsal premotor cortex (CRB vs. PMd) showed increased activations at the same locations and at the same significance levels with only minimal differences (not shown).

At baseline, finger tapping of the right hand was associated with brain activation in the right cerebellar hemisphere and left motor cortex region across groups. Activation of the right lateral CRB was significantly increased in CD patients as compared to healthy controls (Figure 2A, MNI152 coordinates X 21, Y−54, Z−18). Following bilateral cerebellar cTBS, this increased activation was even more pronounced in CD patients (Figure 2B, MNI152 coordinates X 19, Y−59, Z−16). Two other significantly increased activations were found adjacent to the gyrus angularis (MNI152 X−57, Y−42, Z 21) and adjacent to the postcentral sulcus (MNI152 X−55, Y−27, Z 48, Figure 2C). Comparison within the patient group (cTBS on CRB vs. baseline or vs. cTBS on PMd) also showed these elevated activations at the same locations and at the same significance levels with only minimal differences. In contrast, bilateral cerebellar cTBS had no significant effect on brain activation in healthy controls, and PMd stimulation had no effect on tapping-related fMRI activation in both groups. Continuous monitoring of motor performance (timing, duration and frequency) did not reveal significant correlation between groups or stimulation sites.

### TMS

**Figure 3:**
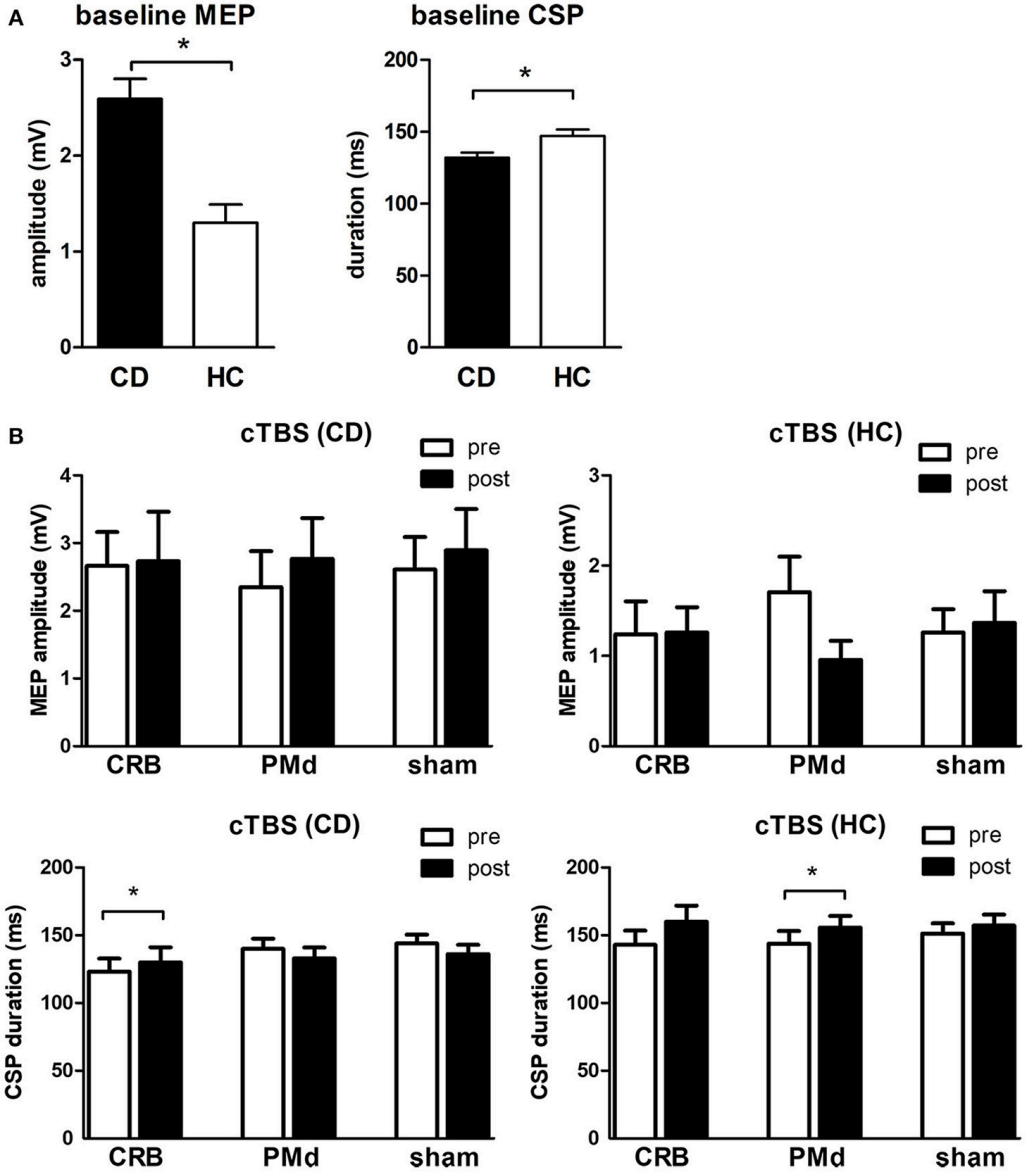
Transcranial magnetic stimulation data**. (A)** Baseline mean motor-evoked potential (MEP) amplitudes and cortical silent period (CSP) duration in CD patients (CD) vs. healthy controls (HC). **(B)** Mean MEP amplitudes and CSP duration before and after cTBS at the cerebellum (CRB), dorsal premotor cortex (PMd), and sham in CD patients and healthy controls. ^*^ indicates significant difference.

At baseline, MEP amplitudes were significantly higher (2.6 ± 1.4 vs. 1.3 ± 1.0 mV, *p* = 0.002) and CSP duration significantly lower (132 ± 23 vs. 147 ± 26 ms, *p* = 0.036) in CD patients as compared to controls (Figure 3A). RMT was lower in CD patients (54.5 ± 16.7 vs. 65.5 ± 12.1, *p* = 0.019), while AMT was comparable between groups (*p* = 0.216). In CD patients, repeated measures two-way ANOVA with the factors STIMULATION MODE [PMd, CRB, sham] and TIME [pre, post] revealed a significant effect of the factor TIME [*F*_(1, 21)_ = 19.59, *p* = 0.0002] on CSP duration. *Post-hoc* analysis showed a significant increase of CSP duration following CRB stimulation (123 ± 27 ms vs. 130 ± 29 ms; adjusted *p* = 0.004), but not after PMd or sham stimulation (Figure 3B). In controls, repeated measures two-way ANOVA with the same factors revealed a significant effect of the factor TIME [*F*_(1, 21)_ = 5.565, *p* = 0.028] and a significant interaction effect of STIMULATION MODE x TIME [*F*_(2, 21)_ = 3.636, *p* = 0.044] on CSP duration. *Post-hoc* analysis showed a significant increase of CSP duration following PMd stimulation (143 ± 30 ms vs. 160 ± 34 ms; adjusted *p* = 0.006), but not after CRB or sham stimulation (Figure 3B). cTBS did not have a significant effect on MEP amplitudes, neither in CD patients, nor in controls (Figure 3B).

### Clinical Assessment

There were no significant changes of TWSTRS and Tsui scores following cTBS at PMd (TWSTRS −1.4 ± 2.0, *p* = 0.146; Tsui −0.3 ± 1.8, *p* = 0.837), at CRB (TWSTRS −0.2 ± 2.7, *p* = 0.816; Tsui −0.4 ± 1.1, *p* = 0.746), or sham stimulation (TWSTRS −0.8 ± 2.4, *p* = 0.705; Tsui + 0.2 ± 0.9, *p* = 0.898). Similarly, CGI-I remained stable after cTBS at any site.

## Discussion

The present study assessed the role of CRB in CD. Employing a multimodal approach comprising functional MR imaging, neurophysiological assessment, and blinded clinical rating, we found CD to be associated with increased brain activation during movement of the (clinically non-dystonic) right hand. Moreover, CD involves an impairment of cortical inhibitory mechanisms, as evidenced by a reduction of CSP duration. Cerebellar interference by TMS enhanced overactivation of CRB while it partially normalized cortical disinhibition. In the following, possible implications of our findings will be discussed.

### Finger-Tapping Related Brain Activation in CD

Finger tapping of the right hand was associated with activation of contralateral M1 and ipsilateral CRB both in CD patients and controls. This is in line with a number of previous studies [e.g., (43–45)] and concurs well with common neuroanatomical knowledge. Combined anatomical, physiological, and imaging evidence suggests that voluntary movements are controlled by a network of regions, comprising motor cortex, basal ganglia, thalamus, dentate nucleus, and cerebellar cortex. CRB is commonly accepted to play a major role in motor task planning and coordination, integration of multisensory peripheral input, and feedback generation to the motor cortex.

**Figure 4:**
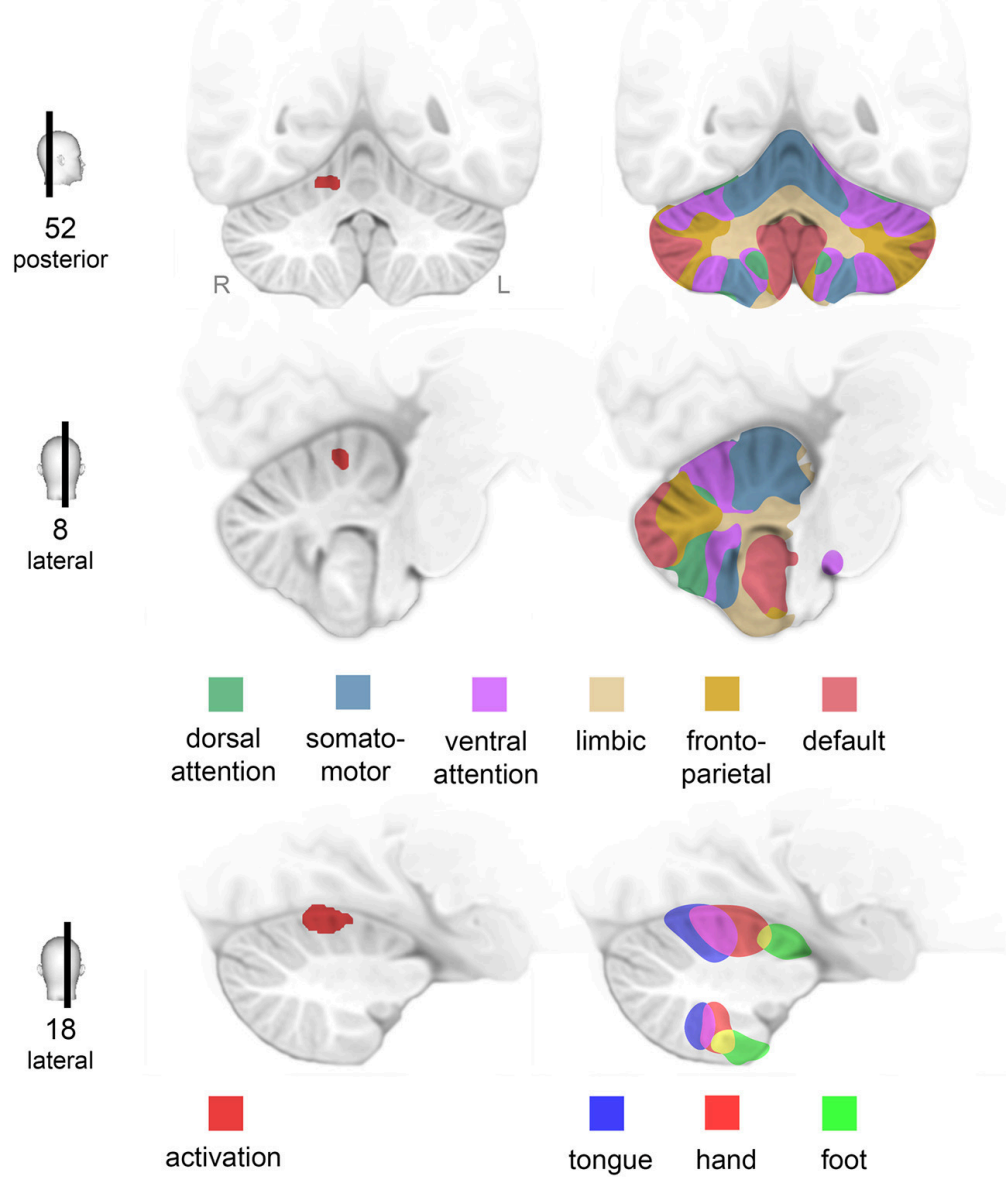
Activation of specific networks in the cerebellum. Finger tapping of the right hand was associated with increased activation (*p* < 0.0001) of the right lateral cerebellum (CRB) in patients with cervical dystonia compared to healthy controls **(middle column)**. Projection on a map of the human CRB based on functional connectivity to seven major brain networks (46) reveals that this area of overactivation is strongly connected to the contralateral hand motor area **(right column)**. Numbers **(left column)** indicate MNI-coordinates (posterior corresponds to y-coordinate, lateral to x-coordinate).

At baseline, activation of the right cerebellar hemisphere was significantly increased in CD patients as compared to healthy controls. The elevated activation was located in the anterior lobe of the CRB. Projection of this area onto a map of the human CRB based on functional connectivity to cerebral networks (46) indicated that this part of the CRB is tightly connected to the hand motor area (Figure 4). Notably, increased cerebellar activation occurred during a simple motor task performed by a non-dystonic limb—a new finding as compared to previous studies in CD patients which did not report cerebellar abnormalities during simple hand motor tasks (19, 20, 47). Both the application of different motor tasks and the use of a scanner with higher magnetic field strength in our study (20) might contribute to this difference.

To interpret our finding, it seems crucial to discriminate reports on abnormalities derived from a clinically dystonic area from findings associated with a non-dystonic movement or even at rest. To our knowledge, only one functional imaging study assessed brain activation during head rotation in CD patients. While isometric (i.e., motionless) head rotation into the direction of the torticollis was associated with an increase of activation in the ipsilateral anterior CRB, isometric rotation into the opposite direction came along with increased activation in ipsilateral precentral and contralateral postcentral cortex regions (22). The authors propose a pathogenic role of the CRB, but compensatory role of the sensorimotor cortex in CD, acknowledging that intentional muscle contraction might differ from involuntary head movements in CD (22).

In contrast, CD patients in the present study were asked to keep their head relaxed while performing a simple tapping task or resting. Within block design, any BOLD signal associated with task-free, CD-related or compensatory muscle contraction was dissolved by subtraction. Thus, cerebellar overactivation can be directly attributed to finger tapping. This may be interpreted as a result of motor overflow, i.e., an unintentional extension of tonic cervical activation into the representations of finger movements, which has become a core feature within the motor phenomenology of dystonic disorders (1, 48). Alternatively—though not mutually exclusively—cerebellar overactivation may be viewed as an indicator of a global “dystonic trait.” Indeed, in a PET study, even completely asymptomatic DYT1 carriers showed increased cerebellar activity at rest (49). Similarly, non-manifesting DYT1 mutation carriers performing at matched levels overactivated the lateral CRB and the right inferotemporal cortex during motor sequence learning compared to age-matched controls (50). Moreover, resting state fMRI revealed an increase of negative cerebello-cortical functional connectivity in patients suffering from writer's cramp who typically are asymptomatic during rest (51). Taken together, one might speculate that cerebellar overactivation during non-dystonic movements or rest may indicate an increased “demand” of tonic cerebellar activity to counter motor cortical overexcitability, well in line with a mainly compensatory role of the CRB (5, 16, 52).

### Cortical Excitability

At baseline, we found higher MEP amplitudes and decreased CSP duration in CD as compared to healthy controls, which is well in line with earlier studies providing evidence of motor cortical disinhibition and concurs with the overall pathophysiological concept of disturbed sensorimotor integration in CD.

Findings about MEP amplitudes in different forms of dystonia are inconsistent, with most studies describing normal (53–58) and only few describing higher (53, 57, 59) amplitudes.

CSP is commonly accepted as a marker of cortical inhibitory capacity mediated by GABAergic transmission (60, 61). Lower CSP duration has already consistently been described in patients suffering from writer's cramp (62), facial (63), and cervical dystonia (57, 64). In CD patients, a positive correlation of CSP duration recorded from the sternocleidomastoid muscle with symptom severity on the TSUI scale was reported, suggesting an impairment of inhibitory motor control to underlie the dystonic symptoms (57, 64). However, as CSP has been assessed remote from a clinically dystonic muscle in the majority of studies, reduced CSP duration may be viewed as another indicator of a global “dystonic trait” in CD patients.

In healthy controls, there was an increase of CSP duration after PMd stimulation. This is well in line with previous data showing reduced M1 excitability after applying this inhibitory protocol to PMd (65), possibly by depression of excitatory connections to M1. Conversely, the lack of an effect of PMd stimulation in the CD group might be interpreted as a further indicator of motor cortical disorganization in dystonia.

### Effects of Cerebellar cTBS on Finger-Tapping Related Brain Activation and CSP

We applied cTBS to the lateral CRB in order to probe the effects of an excitability-modulating protocol on finger tapping related brain activation. We observed even pronounced additional activation of the ipsilateral CRB as well as significantly elevated activation of the contralateral sensorimotor region and the angular gyrus after cerebellar cTBS in CD patients—both compared to healthy controls and compared to baseline and PMd stimulation within the group of patients.

Suprathreshold TMS of the CRB has an inhibitory effect on contralateral M1 excitability, which is usually explained by activation of Purkinje cells leading to an inhibition of dentate nucleus and consequently less excitatory tonic output onto contralateral M1 via dentate-thalamo-cortical connections (34, 66–68). Notably, unilateral cerebellar cTBS, which is performed at subthreshold intensity, has also been shown to decrease contralateral MEP amplitudes (34, 69–71). We therefore suggest that cTBS, rather than directly affecting the Purkinje cells, acts via transsynaptic modulatory effects on stellate and basket cells or parallel fibers within superficial layers of the CRB. As superficial layer cells are known to have inhibitory influence on Purkinje cells, cTBS-induced depression of these cells would eventually result in an inhibition of M1 excitability (34, 70, 72).

It remains open whether activity dependent metaplastic effects, which have been shown to occur at M1 following muscle contractions prior to cTBS (73, 74), might also play a role at cerebellar stimulation. To this end, future studies in healthy subjects will need to disentangle the complex interplay of parameters with potential impact on the net effects of cerebellar cTBS, including motor activity and per interventional head position (58), respectively.

Following cTBS at CRB, we found a significant increase of CSP duration in CD patients. Given shortened CSP at baseline, this may indicate normalization of inhibitory mechanisms acting on M1 by a virtual lesion at the cerebellar hemispheres. The lack of an effect of cerebellar stimulation on CSP in the control group indicates differences between CD patients and controls in respect of their susceptibility to cerebellar “virtual lesions.”

Application of cTBS to bilateral (as opposed to unilateral) CRB in our study confines direct comparison to a small number of previous studies (26, 70, 75). Indeed, CSP did not change following unilateral cerebellar cTBS in CD patients (26), in PD patients (75), nor in healthy subjects (26, 70, 75).

### Clinical Outcome

We did not detect significant effects of cTBS on blindly-rated symptom severity of CD, irrespective of the target site. A simple explanation might be that the impact of a single session of cTBS on the motor network is just too weak to provoke obvious clinical effects, e.g., due to network redundancy (76) and/or fast adaptive mechanisms (77). Our finding is here in line with comparable approaches using single session TBS (78). Notably, previous studies which reported clinical improvement of CD have applied at least 10 sessions of TBS (26, 27). Another reason might be a lack of sensitivity of our rating scales (TWSTRS, Tsui) for small clinical changes. Furthermore, it must be acknowledged that the aforesaid studies (26, 27) used the TWSTRS total score, while we exclusively collected the TWSTRS motor subscale. For instance, interventional effects on the pain subscale, as reported by Bradnam et al. (27), might have contributed significantly to changes of the TWSTRS total score. Finally, potential clinical effects were only assessed once, immediately after the intervention. Bearing in mind the delayed effects of deep brain stimulation in dystonia, it cannot be ruled out that protracted effects of cTBS have escaped our attention.

The fact that two protocols of TBS with opposite effects on the primary motor cortex, applied daily for 2 weeks, previously improved CD symptoms (26) might reveal that their global input on the network disorder itself is quite the same in spite of manifold local effects on cerebellar cortical structures (27). In view of the complex functional network of activating and inhibiting connections, parallel fiber and Purkinje cell interplay, and their dependency on climbing fiber activity, effects of interference by non-invasive stimulation of the cerebellar cortex is obviously hard to predict. A focal effect of TBS on one type of cerebellar neurons may therefore remain an over-simplified view.

## Limitations of the Study

While our subgroups are rather small, they are comparable to previous physiological studies on CD (22, 26, 27), and group size has proved sufficient to show significant differences of brain activation and of physiological measures between groups. It cannot be ruled out, however, that small group sizes contributed to the lack of a significant TMS effect on the clinical scales of CD.

Another limitation might be a potential influence of previous neurotoxin treatment on our neurophysiogical and clinical data in spite of the fact that the experiments were performed at least 10 weeks after the last injection. While an even longer interval between drug application and experimental sessions would have been preferable, this has not been possible both for ethical reasons and for the sake of patient recruitment.

## Conclusion

According to our multimodal approach, interpretation of fMRI data may benefit from physiological and/or clinical input. Given the lack of behavioral changes, the neurophysiological arm of the study may prove most useful to interpret the present findings: CSP, an established measure of cortical inhibitory capacity (79), was reduced in CD patients at baseline, but significantly increased toward normal duration following cerebellar stimulation. In other words, cerebellar cTBS may have partially restored the inhibitory net influence of the CRB on M1 within the cerebello-thalamo-cortical network. On fMRI, we found increased cerebellar activation during simple finger movements in CD patients compared to controls, which were even enhanced by cerebellar cTBS.

Altogether, we interpret our findings in favor of a compensatory role of the CRB within a network disorder underlying CD: If cerebellar overactivation during non-dystonic finger movements indicate a higher “demand” of tonic cerebellar activity to countervail overexcitability of the motor cortex, an indirect, inhibitory net effect of cTBS on Purkinje cells may be able to enhance both cerebellar activation and M1 inhibition. This interpretation, though partly speculative, allows several predictions about measures of cortical excitability/inhibition and cerebello-cortical interactions which can be assessed systematically by future studies.

In conclusion, our combined approach of TMS and fMRI supports the hypothesis of general motor disorganization in CD, which remains subclinical in most body regions and therefore may be characterized a “dystonic endophenotype.” Effects of non-invasive cerebellar interference point to a predominant compensatory function of cerebellar overactivation, which may act as a counterbalance of cortical disinhibition, a core feature of dystonic network disorders. Further research is needed to separate the specific contributions of the CRB in the control of dystonic vs. non-dystonic movements and to disentangle its complex interplay with basal ganglia circuits and the somatosensory system in the range of dystonic disorders.

## Author Contributions

DZ and JV designed the study protocol. DZ, TO, and GH planned the experiments. TO and GH carried out the study. DZ and JV advised on interpretation and analysis of the results. DZ, TO, and GH prepared the first draft of the manuscript. MR and JV reviewed the manuscript.

### Conflict of Interest Statement

The authors declare that the research was conducted in the absence of any commercial or financial relationships that could be construed as a potential conflict of interest.

## Abbreviations

AMT: active motor threshold
BOLD: blood oxygen level dependent
CD: cervical dystonia
CGI-I: Clinical Global Impression Improvement subscale
CRB: cerebellum
CSP: cortical silent period
CTRL: control group
cTBS: continuous theta-burst stimulation
EMG: electromyography
FDI: first dorsal interosseous muscle
fMRI: functional magnetic resonance imaging
iTBS: intermittent theta-burst stimulation
M1: primary motor cortex
MEP: motor-evoked potential
PD: Parkinson's disease
PMd: dorsal premotor cortex
RMT: resting motor threshold
rTMS: repetitive transcranial magnetic stimulation
SD: standard deviation
TWSTRS: Toronto Western Spasmodic Torticollis Rating Scale.
